# ASGR2 and CLEC12A as Prognostically Relevant C-Type Lectin Hubs in Glioblastoma

**DOI:** 10.3390/ijms27062626

**Published:** 2026-03-13

**Authors:** Angelica Pace, Caterina Alfano, Luca D’Angelo, Chiara Napoletano, Ilaria Grazia Zizzari, Antonio Santoro, Marianna Nuti, Lorenzo Farina, Manuela Petti, Aurelia Rughetti

**Affiliations:** 1 Department of Experimental Medicine, Sapienza University of Rome, Viale Regina Elena 324, 00161 Rome, Italy; angelica.pace@uniroma1.it (A.P.); caterina.alfano@uniroma1.it (C.A.); chiara.napoletano@uniroma1.it (C.N.); ilaria.zizzari@uniroma1.it (I.G.Z.); marianna.nuti@uniroma1.it (M.N.); 2 Department of Human Neurosciences, Neurosurgery Division, Sapienza University of Rome, AOU Policlinico Umberto I, Viale dell’Università 30, 00185 Rome, Italy; lucadangelo_80@hotmail.com (L.D.); antonio.santoro@uniroma1.it (A.S.); 3 Department of Computer, Control and Management Engineering, Sapienza University of Rome, Via Ariosto 25, 00185 Rome, Italy; lorenzo.farina@uniroma1.it

**Keywords:** ASGR2, CLEC12A, C-type lectins, immunosuppression, myeloid cells, differential co-expression network, network oncology, glioblastoma

## Abstract

In glioblastoma, the strong immunosuppression of the tumor immune microenvironment fosters tumor aggressiveness and decreases the effectiveness of therapeutic interventions, including immunotherapies. An intricate network of connections among tumor cells, stroma and infiltrating immune cells sustains immunosuppression. Lectins are immunoregulatory glycan-binding receptors contributing to immunosuppression. Their targeting is proposed as an appealing strategy for anti-cancer therapy. In this work, network-based approaches were exploited to identify a lectin profile that could dissect the complexity of tumor-immunity interactions in glioblastoma. Differential co-expression analysis, employing TCGA, CGGA and GTEx databases (145, 133 and 255 samples, respectively), identified a cluster of novel C-type lectins, with ASGR2 and CLEC12A as principal hubs. Furthermore, TIMER2.0 analysis revealed that their expression was significantly associated with immunosuppressive cells. ASGR2 and CLEC12A expression was also validated by cytofluorimetric analysis on both tumor and liquid biopsies from 20 glioblastoma patients. We report that ASGR2 and CLEC12A C-type lectins are associated with tumor-infiltrating immunosuppressive myeloid subsets and discriminate patients’ poor prognosis. These results suggest that C-type lectins may contribute to the immunosuppressive network sustained by infiltrating myeloid immune cells in GB, resulting in exploitable targets for therapeutic interventions.

## 1. Introduction

**Figure 1:**
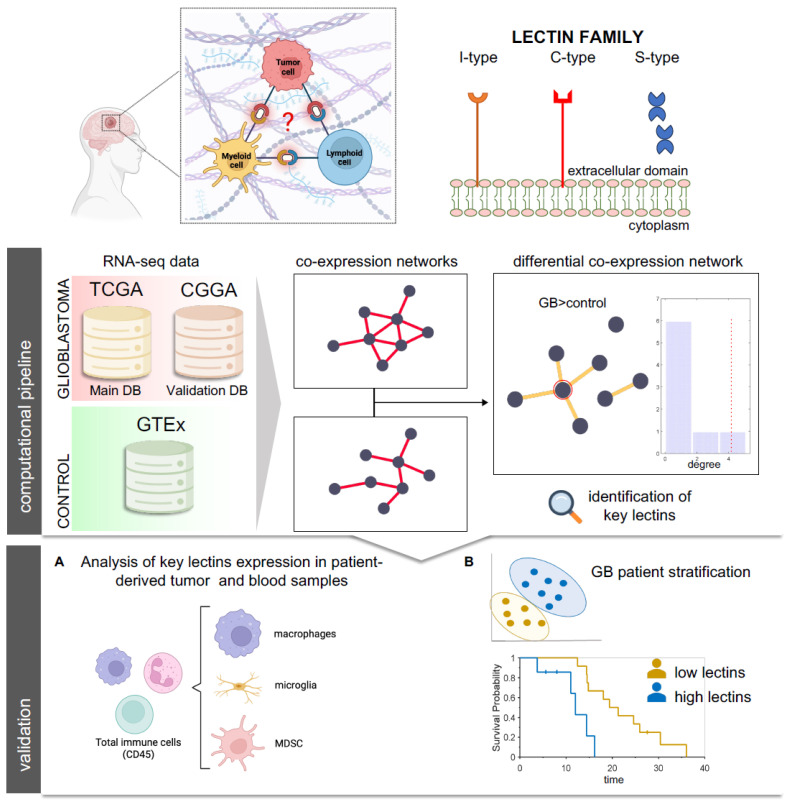
Identification of novel lectins and lectin networks involved in the immunoregulatory circuits acting within the GB microenvironment. In the computational pipeline phase, RNA-seq data of the lectin members belonging to galectin, C-type and Siglec lectin families were selected and screened from TCGA, CGGA and GTEx databases (DB), and differential co-expression networks were built (computational pipeline). In the validation phase, the expression of the novel identified lectins was assessed in GB-infiltrating myeloid immune cells by flow-cytometry (**A**) and the prognostic significance of the lectin network was investigated (**B**). In part created with BioRender, https://BioRender.com/2s5agwx; https://BioRender.com/ga28q8t (accessed on 20 February 2026). TCGA: The Cancer Genome Atlas Program; CGGA: Chinese Glioma Genome Atlas; GTEx: Genotype-Tissue Expression.

Glioblastoma (GB) is the most common and aggressive brain tumor in adults. With the latest brain tumor classification released by the World Health Organization (WHO) in 2021, GB is now identified as isocitrate dehydrogenase (IDH)-wildtype [1]. Today the standard of care at first diagnosis requires maximal safe resection followed by chemotherapy and radiotherapy treatment [2]. Despite the latest advances in understanding tumors’ molecular and genetic complexity and the novel therapeutic strategies that have been proposed, no efficacious improvement in therapeutic GB management has been made [3,4,5,6]. The immunosuppressive microenvironment and its interplay with altered angiogenesis, hypoxia and oxidative stress guide GB aggressivity and resistance to standard as well as more innovative immunotherapy approaches [7,8,9,10]. In GB, alterations in blood–brain barrier (BBB) integrity due to microvascular sprouting, new vasculature branching and dysfunction of tight junctions allow the recruitment and infiltration of inflammatory cells, such as macrophages and myeloid-derived suppressor cells (MDSCs), that play a crucial role in fostering immunosuppressive circuits and enforcing the hypoxic niche, thus metabolically reprogramming the tumor immune microenvironment (TIME) components [11,12,13]. Tumor-associated macrophages (TAMs) constitute the major infiltrating myeloid population in the GB microenvironment [14]. TAMs are highly immunosuppressive and promote extracellular matrix (ECM) remodeling, sustain tumor cell proliferation and migration and recruit other immunosuppressive cells, such as MDSCs and regulatory T cells (Tregs) [14,15]. Given their prominent role in GB immunosuppression, TAMs have been associated with the worst prognosis and progression of GB and have been proposed as potential therapeutic targets [16,17,18,19]. MDSCs sustain GB by releasing arginase 1 (ARG1) and reactive oxygen species (ROS) and inhibiting CD3^+^ T cells [12,20,21,22,23]. Moreover, high levels of both circulating and infiltrating MDSCs have been associated with a worse prognosis in GB patients [24,25]. Furthermore, the resident microglia within the tumor undergo functional changes, thus contributing to immunosuppression [14,26,27,28]. Dismantling these immunoregulatory intersected networks is the keystone required to allow therapeutic approaches to be effective. On the other hand, the distinct and synergistic molecular mechanisms and biological players that cause the inter- and intratumor heterogeneity of the GB immune landscape have yet to be unveiled [29,30,31]. In this study, we aim to investigate the repertoire of lectin receptors relevant to GB by employing network analysis. Lectins are glycan-binding receptors mainly expressed by immune cells and recognize specific glycosylated motifs through specialized recognition domains (CRDs) [32]. They tightly modulate a large variety of physiological processes, including immune responses. In cancer, lectin-mediated interactions trigger immunoregulatory switches. For this reason, they can serve as biomarkers of disease and prognosis and be exploited as therapeutic targets to hamper and weaken cancers, while reinforcing the anti-tumor immune response [33,34]. In GB, few studies report that lectin expression is associated with disease aggressiveness and immunosuppression, and evidence is mainly related to the galectin and sialic binding lectin (Siglecs) receptors. GB tumor cells express galectins (-3, -8, -9), lectins recognizing β-galactoside, which are correlated with the aggressiveness of the disease [35,36]. Siglecs, recognizing sialic acid, are expressed by infiltrating and circulating MDSCs in GB patients, and inversely correlate with prognosis [37,38,39]. Siglec-9 appears to impact GB-infiltrating macrophage functions and to play a crucial role in GB immunosuppression [38,40]. On the other hand, little is known about the expression of C-type lectins [35]. However, these studies focus on a single lectin or the interpretation of differences in the abundance of lectin transcripts without considering the presence of active patterns of interconnections among them. To identify novel lectins possibly contributing to GB immunosuppression networks, we employed publicly available RNA-seq datasets (from both tumor and normal brain tissues) and network-based approaches, since they represent powerful tools for dissecting the high complexity of biological systems and investigating physiological and pathological molecular mechanisms [41,42,43,44]. Regarding gene expression data, co-expression networks highlight genes that tend to show a similar expression profile that can be considered associated with common biological processes [45]. A further level of investigation concerns the study of differential co-expression networks. These networks identify alterations in gene co-expression patterns observed in two different conditions, with the main aim of detecting a sub-network or a set of relevant genes that operate abnormally and affect a significant number of neighbors across sample groups [46,47]. In this study, we focus on identifying differentially co-expressed lectins among Siglecs, galectins and C-type lectins and on validating the expression and clinical relevance of novel key GB-relevant lectins for potential targeting (Figure 1).

We identified a novel cluster of C-type lectins with ASGR2 and CLEC12A as principal hubs, never described before in glioblastoma, and validated their expression in tumor and blood samples, and then correlated them with patients’ survival. These results suggest that novel C-type lectins may participate, together with Siglecs and galectins, in the maintenance of the GB immunosuppressive network, thus identifying these receptors as possible therapeutic targets.

## 2. Results

### 2.1. Differential Co-Expression Analysis Reveals ASGR2 and CLEC12A as Novel Key Lectins in GB Patients

**Figure 2:**
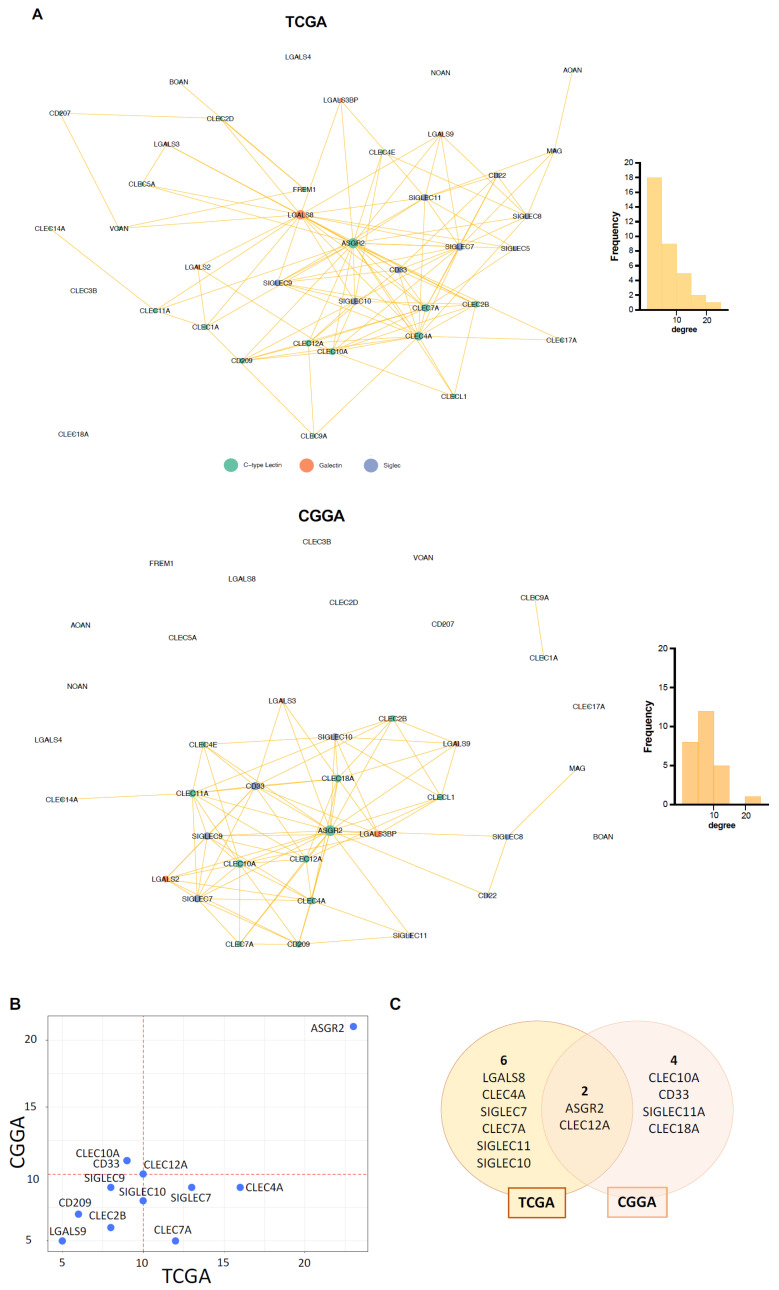
ASGR2 and CLEC12A: Novel C-type lectins identified by differential co-expression network analysis. (**A**) At the top is the TCGA vs. GTEx differential co-expression network, while at the bottom is the CGGA vs. GTEx differential co-expression network. Node color codes for the lectin family and the links represent a significant increase in co-expression between two lectins in GB with respect to the healthy condition. (**B**) Scatter plot showing significant correlation between the lectins’ degrees in the two differential networks: only lectins involved in at least 5 differential links are shown. (**C**) Venn diagram showing the degree of agreement between the two differential networks with respect to the sets of hub lectins.

To investigate lectins that statistically increase their co-expression in GB compared to levels in the healthy brain, we constructed a differential co-expression network. Here, the Spearman correlation coefficients for all possible pairs among the 39 selected lectins were calculated as a measure of co-expression for each dataset. Then, after applying Fisher’s *Z*-transformation on the Spearman coefficients, the difference in correlation between tumor samples and the control cohort was evaluated by computing Z-scores. The TCGA-GTEx differential network is shown in Figure 2A (upper panel). By analyzing this network, we identified eight hub lectins, thus inferred to operate abnormally in GB and to affect a significant number of neighboring lectins. These lectins are: ASGR2, LGALS8, CLEC4A, SIGLEC7, CLEC7A, SIGLEC10, SIGLEC11, and CLEC12A. It is worth noting that the three lectins with the highest degrees (ASGR2, LGALS8 and CLEC4A) are not differentially expressed, while the other hubs are upregulated (Supplementary Table S1). Interestingly, C-type lectins are mainly represented in the hub group (ASGR2, CLEC4A, CLEC7A, CLEC12A vs. LGALS8 vs. SIGLEC-7, -10, -11). Similar results were obtained when analyzing the CGGA-GTEx differential network (Figure 2A, bottom panel). Indeed, the lectin rankings based on the degree in the two networks appear to agree (Spearman correlation = 0.6, Figure 2B). It is important to note that among the lectins with the highest degree of agreement between TCGA and CGGA, we identified lectins that have already been described as being expressed and playing a role in human GB, such as SIGLEC7, SIGLEC9 and LGALS9, thus indicating adequate correspondence of our network analysis to biological data already available [35]. Therefore, it is striking that two undescribed C-type lectins, i.e., ASGR2 and CLEC12A, were two main hubs shared by both databases (Figure 2C).

### 2.2. ASGR2 and CLEC12A Are Associated with Survival and Immune Infiltrate in GB

**Figure 3:**
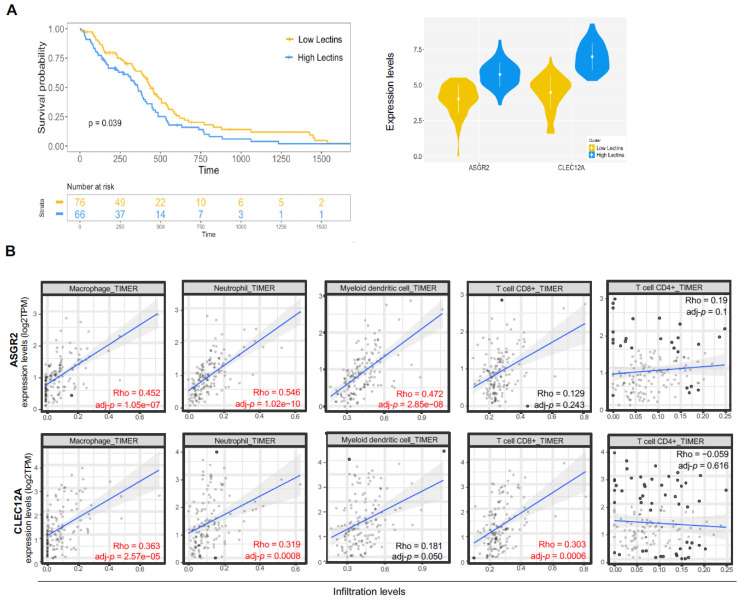
ASGR2 and CLEC12A correlation with survival and immune infiltrating cells in GB patients. (**A**) Kaplan–Meier survival curves of GB patients distributed in the two patient groups [Low lectins (76 patients) vs. High lectins (66 patients)] based on ASGR2-CLEC12A combined expression. A log-rank test was employed to determine the statistical significance of the observed difference (*p* = 0.039) (left panel). Expression levels of ASGR2 and CLEC12A in the two patient groups (right panel). (**B**) Scatter plots represent the correlation between ASGR2 and CLEC12A expression (log2TPM) and the infiltration levels of macrophages, neutrophil, myeloid dendritic cell, and CD8^+^ and CD4^+^ T cell estimated by the TIMER algorithm in GB using the TIMER2.0 tool. For each correlation, Rho and adjusted *p* values are indicated.

Given their important role in co-expression changes between tumor and healthy samples, confirmed by both databases, we asked whether the two lectins could serve as markers of survival for GB patients. Considering the TCGA-GB cohort, we used a soft clustering algorithm and identified two groups of patients based on ASGR2 and CLEC12A alone or their combined expression (Figure 3A).

ASGR2 and CLEC12A alone did not correlate with GB patients’ survival (Supplementary Figure S1). When patients were stratified based on ASGR2 and CLEC12A combined expression, patients with low levels of both lectins had a significantly improved survival expectancy compared to patients with high levels (*p* = 0.039) (Figure 3A). No differences in terms of clinical parameters between the two groups were observed (Supplementary Figure S1), underlining that there were no clinical biases that could influence the different survival outcomes. We then evaluated the association of each lectin with infiltrating immune cell subsets, taking into consideration the heterogeneity of GB TIME composition (Figure 3B). We calculated ASGR2 and CLEC12A correlations with macrophages, neutrophils, myeloid dendritic cells, and CD8^+^ and CD4^+^ T cells estimated with the TIMER algorithm. As shown in Figure 3B, ASGR2 and CLEC12A expression levels positively correlated with infiltration levels of all three myeloid cell subsets, suggesting their putative contribution to immunosuppressive mechanisms. CLEC12A also displayed a positive correlation with CD8^+^ T cells, which in the GB TIME are usually found in an exhausted state.

### 2.3. ASGR2 and CLEC12A Are Expressed by Infiltrating Myeloid Cells in GB

**Figure 4:**
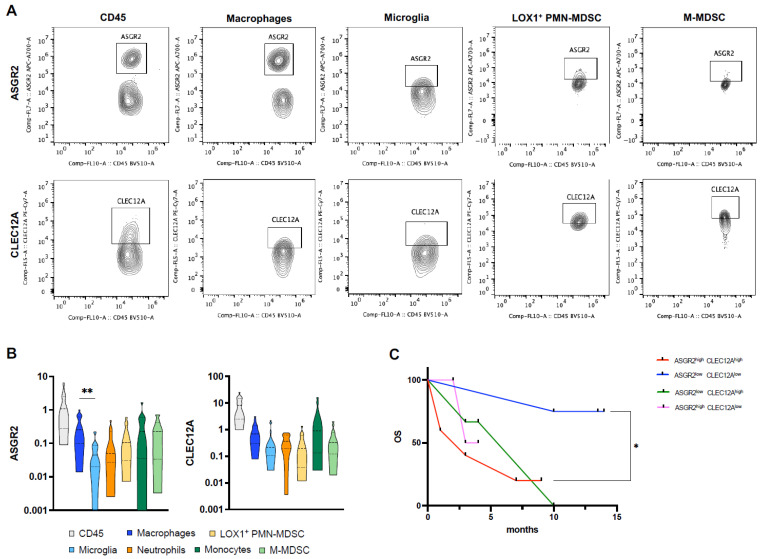
ASGR2 and CLEC12A expression on human GB-infiltrating myeloid cells and correlation with clinical outcome. (**A**) Dot-plot of representative patient-derived tumor sample analysis of ASGR2 and CLEC12A expression on total immune cells (CD45) and macrophage, microglia, neutrophil, monocyte and MDSC subsets. (**B**) Histograms represent the mean ± SEM of ASGR2 and CLEC12A expression on infiltrating immune cell subsets (macrophages, microglia, LOX1^+^ PMN- and M-MDSC) from 20 GB patients. Data are reported as percentage of CD45^+^ total infiltrating immune cells. ANOVA followed by Tukey’s multiple comparison test was used for comparison between groups; **, *p* = 0.00949. (**C**) Kaplan–Meier curves of GB patients based on the combined expression of ASGR2 and CLEC12A in infiltrating CD45^+^ cells. Differences between the Kaplan–Meier curves of OS were assessed using the log-rank test. *, *p* = 0.0249.

So far, no studies have described the expression of ASGR2 and CLEC12A in GB, while their expression has been recently reported in more aggressive disease in gastric cancer and acute myeloid leukemia (AML), respectively [48,49,50]. To validate the presence of these two lectins revealed by differential network analysis in GB, we characterized their expression on infiltrating immune cells freshly isolated from 20 patients’ GB tumor samples (Figure 4).

The specific infiltrating myeloid cell subsets relevant to immunosuppression [resident microglia (MG) and infiltrating macrophages, monocytic and polymorphonuclear MDSCs (M-MDSC and PMN-MDSC, neutrophils, monocytes)] were analyzed (Supplementary Figure S2). It is widely recognized that high infiltration of immunosuppressive cells in GB negatively impacts patients’ prognosis [51]. Not only the different immune landscape plays a crucial role in GB progression, but the difference in receptor expression on specific immune cells could also contribute to the immunosuppressive mechanism and resistance to therapy. We characterized the distribution of ASGR2 and CLEC12A on these specific immune myeloid cell subsets (Figure 4A,B). Tumor-infiltrating macrophages exhibited higher ASGR2 levels than resident microglia (mean ASGR2 macrophages vs. microglia: 0.34 ± 0.06 vs. 0.06 ± 0.01, *p* = 0.0094) (Figure 4B). CLEC12A is expressed by each myeloid immune cell subset with no significant differences among them (Figure 4B). To evaluate the prognostic role of ASGR2 and CLEC12A and validate computational analysis in our cohort, we stratified patients based on median ASGR2 and CLEC12A expression on CD45^+^ cells. We identified four groups of patients: (i) ASGR2^high^CLEC12A^high^, (ii) ASGR2^high^CLEC12A^low^, (iii) ASGR2^low^CLEC12A^high^, (iv) ASGR2^low^CLEC12A^low^. The Kaplan–Meier curves show that only patients with low levels of both ASGR2 and CLEC12A displayed increased OS compared to those with high levels, validating computational analysis (HR: 6.678, 95% CI: 1.092 to 40.84, *p* = 0.0249) (Figure 4C).

### 2.4. ASGR2 and CLEC12A Display a Distinct Distribution Profile on Circulating Myeloid Cells

**Figure 5:**
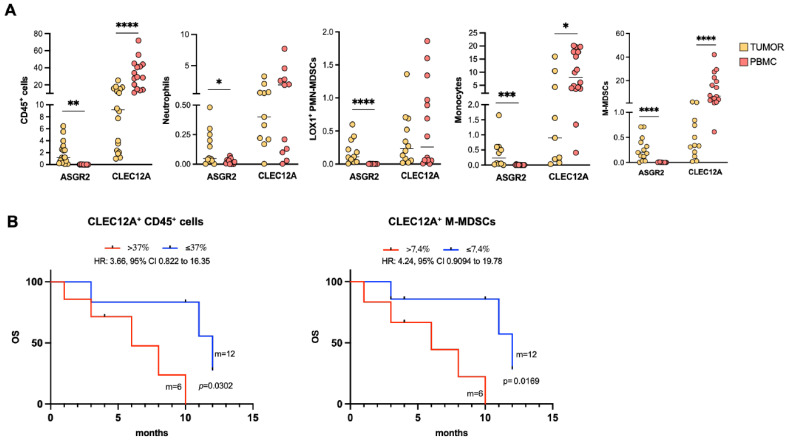
ASGR2 and CLEC12A expression in circulating myeloid cells and correlation with survival. (**A**) Scatter dot-plots present ASGR2 and CLEC12A distribution on circulating myeloid cells (CD45^+^, neutrophils, monocytes, LOX1^+^ and M-MDSCs) and their respective infiltrating subsets. Paired Student *t*-test was used to compare two groups and *p* values < 0.05 were considered statistically significant [ASGR2-tumor vs. ASGR2-PBMC: CD45^+^ (*p* = 0.0014), neutrophils (*p* = 0.0207), LOX1^+^ PMN-MDSCs (*p* < 0.0001), monocytes (*p* = 0.0006), M-MDSCs (*p* < 0.0001); CLEC12A-tumor vs. CLEC12A-PBMC: CD45^+^ (*p* < 0.0001), monocytes (*p* = 0.0120) and M-MDSCs (*p* < 0.0001)], * *p* < 0.05, ** *p* < 0.01, *** *p* < 0.001, **** *p* < 0.0001 (**B**) Kaplan–Meier survival curves of GB patients based on CLEC12A median expression on CD45^+^ and M-MDSCs subsets. Differences between the Kaplan and Meier curves of OS were assessed using the log-rank test [CLEC12A^+^ CD45^+^ cells (>37%) vs. CLEC12A^+^ CD45^+^ cells (≤37%): *p* = 0.0302; CLEC12A^+^ M-MDSCs (>7.4%) vs. CLEC12A^+^ M-MDSCs (≤7.4%): *p* = 0.0169].

These results prompted us to evaluate expression levels and distribution patterns of ASGR2 and CLEC12A in PBMCs (Figure 5A).

Surprisingly the two lectins behaved differently: ASGR2 expression on circulating immune cells was undetectable, while it was well expressed in the matched patient tumor biopsies, suggesting that ASGR2 could be specifically restricted to the tumor-infiltrating macrophagic subpopulation and strictly associated with underlying immunosuppressive mechanisms and molecular features of the GB TIME. On the other hand, CLEC12A was found in all circulating myeloid cell subsets, with strong expression in those with a monocytic origin, also reflecting its distribution on infiltrating myeloid cells (Figure 5A). Interestingly, CLEC12A expression was significantly higher on circulating CD45^+^ cells, monocytes and M-MDSCs compared to their respective infiltrating subsets (*p* < 0.0001, *p* = 0.0120, *p* < 0.0001, respectively). Interestingly, CLEC12A emerged as a potential circulating biomarker of survival (Figure 5B). Indeed, the Kaplan–Meier curves show that high levels of CLEC12A^+^ CD45^+^ cells, particularly CLEC12A^+^ M-MDSCs, negatively correlated with OS (CLEC12A^+^ CD45^+^ > 37% vs. CLEC12A^+^ CD45^+^ ≤ 37%: 6 months vs. 12 months; HR: 3.66, 95%CI: 0.822 to 16.35, *p* = 0.0302; CLEC12A^+^ M-MDSCs^+^ > 7.4% vs. CLEC12A^+^ M-MDSCs^+^ ≤ 7.4%: 6 months vs. 12 months; HR: 4.24, 95%CI: 0.9094 to 19.78, *p* = 0.0169).

## 3. Discussion

Despite therapeutic improvements in the era of immuno-oncology, GB remains one of the most aggressive tumors to treat. The high recurrence rate, the low survival and the poor quality of life underline the urgent unmet need for efficacious, novel therapeutic treatments. One of the key mechanisms that guide GB progression and resistance to therapy is hypoxia. Hypoxia promotes the alteration of the vasculature and guides immunosuppression by enhancing the immunosuppressive function of infiltrating macrophages and myeloid cells [12,52]. Changes in glycosylation also occur under hypoxic conditions and lead to the generation and modulation of *N*- and *O*-glycans, such as Tn antigen, that could modulate the local and distant immune response, thus representing putative therapeutic targets [32,53,54]. Lectins are glycan-binding receptors that have been described and associated with immunosuppressive mechanisms and inhibition of anti-tumor immune responses in different tumor histotypes [32,53,54]. Due to their tightly and highly specific regulated functionality, their targeting has been proposed as an appealing therapeutic strategy to selectively interfere with immune balance [55,56]. To date, Siglecs (-5, -7, -9) and galectins (gal-1, -3, -8, -9) have been mainly described in GB, while C-type lectins have been poorly investigated [35]. Here, we aimed to identify the C-type lectins, Siglecs and galectins that could be involved in GB by exploiting network-based approaches through the analysis of TCGA and GTEx databases, using CGGA as a validation dataset. Network analysis is fundamental to dissecting the complexity of tumors and identifying not only the actors in specific networks but also the relationships among them [41,44,57]. Indeed, the pathological progression could be mediated by de novo factors that are specific to the disease, as well as by novel interactions among mediators, while their expression levels do not change. We found that the lectin co-expression network in GB differs from that in healthy tissue. Even if some of the key lectins are shared with healthy tissue, the interactions among them in GB are different and could have an impact on tumor development. To better understand the relevant interactions in GB, we built a differential co-expression network between tumor (TCGA) and healthy tissue (GTEx). Interestingly, the differential lectin network included hub genes that showed no increase in transcriptomic levels, like ASGR2, LGALS8 and CLEC4A (Supplementary Table S2); however, their interactions with other lectins in the network changed and represent putative underlying mechanisms. This highlights how a network-based approach allows for a focus on molecules and interaction mechanisms that would have been ignored with a standard approach based only on changes in the expression levels of individual molecules. Strikingly, the identified differential network contained novel C-type lectins as prevalent hubs (ASGR2, CLEC12A, CLEC4A, CLEC7A), together with lectins already known to be involved in GB aggressiveness and immunosuppression, such as Siglecs (Siglec-7, -11, -10) and galectin-8 [35]. Our findings were also validated by differential co-expression analysis, performed employing the CGGA and the GTEx datasets. ASGR2 and CLEC12A emerged as principal hubs of differential co-expression networks from both TCGA and CGGA, despite slight differences between the two differential co-expression networks, which may be due to the different ethnicities of the patients listed in the two datasets (Caucasian vs. Asiatic populations) with different impacts on disease incidence and lectin polymorphism [58,59,60]. So far, very little is known about C-type lectins in GB: the C-type lectin CLEC10A has been associated with increased PD-L1^+^ infiltration of immunosuppressive macrophages in a GB mouse model, while CLEC5A has been shown to promote GB progression and immunosuppression and has been associated with poor prognosis [61,62,63]. To the best of our knowledge, no evidence is available about ASGR2 and CLEC12A expression and function in GB. Interestingly, ASGR2 and CLEC12A appear to be involved in brain pathologies such as Alzheimer disease and epilepsy [64,65,66]; on the other hand, their involvement in cancer has been described for other tissue districts, but not GB. CLEC12A, also known as MICL, is an inhibitory C-type lectin that is expressed by myeloid cells, such as monocytes, neutrophils and macrophages, and mainly suppresses inflammatory responses. To date, dead cells, hemozoin and uric acid have been described as CLEC12A ligands [67,68,69]. Dead cells or damaged neurons are removed by microglia, the resident myeloid cells that surveil brain parenchyma and sustain tissue homeostasis [70]. The dysregulation in apoptotic pathways is a critical feature of GB TME, sustained by hypoxia and altered vasculature, leading to the activation of pro-inflammatory microglia and recruitment of immunosuppressive myeloid cells that sustain tumor progression [70,71,72]. Based on concentration and context, uric acid has a dual role in brain health: it serves as an antioxidant, offering neuroprotection, or promotes inflammation, sustaining neurodegenerative disease and cognitive dysfunction [73,74]. In cancer, high levels of uric acid correlate with disease aggressivity and poor prognosis. Changes in uric acid levels are associated with purine metabolism alterations that appear to sustain GB cells’ self-renewal and growth [75]. In cancer, CLEC12A has been mainly studied in AML, where it is aberrantly expressed and represents a biomarker to assess minimal residual disease and leukemic stem cells phenotype, but no evidence in GB is reported [76]. However, CLEC12A has been described to be involved in neuroinflammatory diseases. In a recent study in a murine model of neuroinflammation and encephalitis, CLEC12A deficiency was accompanied by increased T-cell recruitment and activation in the brain, along with the upregulation of antigen-presenting genes and pro-inflammatory cytokines, suggesting that CLEC12A-mediated signaling impairs protective immune responses [77]. In an experimental autoimmune encephalomyelitis (EAE) mouse model, the authors identified CLEC12A as a key receptor for dendritic cells (DCs) trafficking into the CNS, where they sustain disease progression. Blocking CLEC12A resulted in decreased infiltration of DCs and disease severity [78]. Similar effects were observed in EAE mouse models, where treatment with apigenin, a natural flavonoid, was accompanied by reduced CLEC12A expression and prevented demyelination and immune cell infiltration [79]. Moreover, in murine models the selective targeting of CLEC12A stimulated both CD4^+^ and CD8^+^ T-cell proliferation [80]. To date, no direct evidence links CLEC12A with the GB TME. However, the association of CLEC12A with infiltrating myeloid cells and data in neurodegenerative disease may support the hypothesis that this pathway may be relevant in GB immunosuppressive mechanisms. ASGR2 recognizes *O*-glycans β-D-galactose and N-acetylgalactosamine residues in desialylated glycoproteins [32]. *O*-glycans are fundamental for neuronal development and functions [81]. Increasing evidence highlights alterations in *O*-glycosylation pathways as a key feature in neurodegenerative disease and glioma [81,82]. ASGR2 has been recently studied and associated with poor prognosis, recurrence and resistance to treatments for gastric cancer [49]. In gastric cancer mouse models, after prolonged polystyrene exposure, increased ASGR2 expression was accompanied by increased cancer hallmarks, such as PD-L1 and N-cadherin, proliferation, migration and invasive ability [83]. Moreover, a recent study identified ASGR2 as a biomarker related to macrophage lactylation in chronic obstructive pulmonary disease (COPD), highlighting its relevance in immune microenvironment and metabolic pathway modulation [84]. In brain diseases such as Parkinson’s disease (PD), ASGR2 emerged as a key upregulated gene involved in necroptosis and strictly associated with M2 macrophages, while we recently highlighted that histone lactylation boosts immunosuppressive functions of macrophages in GB [11,66]. In silico analysis exploiting the TIMER2.0 database revealed that both ASGR2 and CLEC12A were significantly associated with myeloid immune infiltration, particularly macrophages and neutrophils, which represent the most prominent immune cell subsets in the GB TIME [85]. CLEC12A also appeared to be strongly associated with CD8^+^ T cells, which in GB are commonly found in an exhausted state and express high levels of inhibitory immune checkpoints, such as PD1, TIGIT, and LAG3, again suggesting that CLEC12A-expressing cells may be involved in immunosuppression [86]. These results were confirmed by a validation in our cohort of GB patients showing that both ASGR2 and CLEC12A were expressed by tumor-infiltrating CD45^+^ immune cells from GB tumor samples. ASGR2 expression is significantly higher on infiltrating macrophages compared to resident microglia, while CLEC12A appears to be expressed in all the infiltrating myeloid subsets. Notably, only the combined expression levels of ASGR2 and CLEC12A correlate with better survival probability, both in the dataset analyzed and in our GB patient cohort, suggesting a synergy between them. Myeloid cells play a crucial role in the intricate and dynamic complexity of the GB microenvironment, and their metabolic reprogramming is crucial for immunosuppression [87]. Lectin synergy may be a biological mechanism involved in this intratumor reprogramming, and we are currently exploring this working hypothesis. Furthermore, our data indicate that ASGR2 expression may represent a selective feature of tumor-infiltrating macrophages and not of myeloid circulating cells, suggesting it might play a role in the TIME. On the other hand, CLEC12A expression is higher on circulating myeloid subsets than on the infiltrating ones. It is particularly expressed by cells of monocytic origin, such as M-MDSCs and monocytes, and associated with a worse prognosis, offering a potential biomarker that needs to be validated in a larger GB patient cohort. Although further biological studies are required to validate this hypothesis, we believe that these results highlight the possible employment of C-type lectins as targets to elucidate immunosuppressive mechanisms, as well as potential immune biomarkers in the design of precision therapeutic strategies for GB and their employment in biomarker development. It is important to note that lectin targeting may be beneficial for characterizing immunosuppressive networks, but given their broad spectrum of expression, it can alter the homeostasis of the immune system and lead to autoimmunity [88]. Thus, interfering with the glycan–lectin axis with glycoconjugates or glycomimetic peptides or implementing the use of bispecific antibodies, fusion proteins or antibody–lectin chimeras may help in the selective targeting of these mechanisms in the TME [55,89,90,91,92,93,94]. Interestingly, a bispecific antibody targeting CLEC12A on AML blasts and CD3 on T cells was able to amplify the T-cell-mediated cytotoxicity directly against cancer cells [95]. Precision oncology has progressively reshaped cancer treatments, and different specific targeted therapies have been employed and extended in several tumor histotypes, including in GB and lectin targeting may be a feasible option [96,97,98]. 

## 4. Conclusions

Here, we identified, by differential co-expression analysis, a group of C-type lectins, Siglecs and galectins that could be relevant in GB. Among them, we described a cluster of C-type lectins, particularly ASGR2 and CLEC12A as major hubs in the network, and characterized their expression on tumor-infiltrating immune cells and in matched circulating immune cells obtained from GB IDH^wt^ patients. Both ASGR2 and CLEC12A are expressed by infiltrating myeloid cells, while CLEC12A is also present on circulating CD45^+^ cells. Their combined expression levels identify patients with worse prognosis, suggesting their possible synergic contribution to the immunosuppressive network established in the TIME. Furthermore, the CLEC12A levels detected in M-MDSCs are associated with worse prognosis. The relevance of the lectin-mediated immunosuppressive network, the lectin binding specificity and the availability of synthetic glycoconjugates make lectin detection and targeting an appealing therapeutic strategy to be further investigated for GB.

## 5. Materials and Methods

### 5.1. Data and Preprocessing

We used public TCGA data repositories (http://cancergenome.nih.gov/, accessed on 1 September 2022) as our primary sources of samples. From the TCGA database we downloaded RNA-seq data and clinical annotations of 152 primary GB multiforme patients (TCGA-GB project). We removed patients with IDH1^+^ mutations, obtaining a final cohort of 145 GB samples. For the purposes of the study, we identified a set of genes coding for the 67 molecules of interest (14 siglecs, 15 galectins and 38 C-type lectins; Supplementary Table S2). Only a subset of them (57 lectins) were available in the TCGA-GB dataset, and therefore, for each patient, we considered the gene expression profile associated with this subset of genes. Filtering was carried out to remove lowly expressed genes. Genes whose total sum across samples was less than 500 counts were filtered out. Therefore, the number of lectins decreased from 57 to 39. Two additional datasets were included in the study. First, the GTEx dataset was identified for the control condition. Gene expression data of 255 samples (deceased adult healthy donors) from the “brain cortex” dataset (Release V8) were downloaded from the GTEX Portal (https://www.GTExportal.org/home/tissue/Brain_Cortex, accessed on 15 February 2023). As a validation dataset, the mRNAseq_693 dataset on the Chinese Glioma Genome Atlas (CGGA, http://www.cgga.org.cn/, accessed on 15 February 2023) was considered. We selected the 109 non-IDH1^+^ mutated patients among the primary GB samples. Filtering to remove poorly expressed genes was also performed in these two additional datasets. Therefore, in the CGGA dataset, 3 lectins did not meet the inclusion criterion (SIGLEC5, CLEC5A and CD207), and therefore, the gene expression profiles were composed of 36 genes instead of 39. The three downloaded transcriptomics datasets were normalized with Deseq2 and log2-transformed. The final number of cases used in the present study is indicated in the Supplementary Table S3.

### 5.2. Differential Expression Analysis

Differential expression analysis was performed comparing cancer and control samples from the TCGA and GTEx databases, respectively. We first computed the magnitude of differential expression, measured by the fold change, i.e., the ratio between the mean expression levels in cancer samples and control samples, on the log2 scale. We then performed a statistical test (*t*-test, false discovery rate correction) to assess the significance of the differential expression. We identified differentially expressed genes based on the following criteria: *abs*(*log*_2_*FC*) ≥ 1.5 and *adj-p* < 0.05.

### 5.3. Differential Co-Expression Network Analysis

For each dataset, we employed Spearman’s rank correlation coefficient to evaluate the level of co-expression among lectins. Based on the hypothesis of the study, we considered only positive correlations, removing all the negative links from the obtained networks (Supplementary Figure S3). Then, to construct the differential co-expression network (TCGA-GB vs. GTEx control group), we applied Fisher’s *Z*-transformation to the correlation coefficients, and, for each gene pair, we computed the z-score as follows:

(1) $$Z=\frac{z_{T}-z_{C}}{\sqrt{\frac{1}{n_{T}-3}+\frac{1}{n_{C}-3}}}$$

where $z_{T}$ and $z_{C}$ are the Fisher *Z*-transformation of, respectively, the correlation between the genes in the tumor and control samples, while $n_{T}$ and $n_{C}$ are the number of samples of each group. We set the z-score threshold to 3 to focus only on the significant increase in co-expression between lectins in GB. For validation purposes, the same procedure (Spearman’s rank correlation, Fisher’s *Z*-transformation of the correlation coefficients and z-score computation) was applied to obtain the differential co-expression network between CGGA samples and the GTEx database. In this case, we set the threshold for the z-scores to ensure that the two differential networks (TCGA, CGGA) were characterized by a comparable density. We identified key nodes (i.e., key lectins) based on the number of links they are involved in, and therefore, the number of significant co-expressions increases in GB. For this purpose, we calculated the degree index: hubs of the differential co-expression networks were identified by selecting the 80th percentile of the nodes’ degree distribution.

### 5.4. TCGA Patient Stratification and Survival Analysis

Patients were clustered based on the expression levels of the top relevant lectins highlighted by differential co-expression analysis. We performed Fuzzy C-means clustering with the R package *ppclust* [99], a soft clustering technique in which each data point is separated into different clusters and then assigned a probability score for being in that cluster; this method is therefore more robust to outliers in comparison to hard clustering techniques, like K-means. Finally, we used the information available in the TCGA dataset to compare the survival outcomes of the identified groups using the R packages *survival* and *survminer* to generate Kaplan–Meier plots and risk tables.

### 5.5. Tumor-Infiltrating Immune Cell Analysis by TIMER 2.0

Tumor Immune Estimation Resource 2.0 (TIMER 2.0) (http://timer.cistrome.org/, accessed on 5 July 2025) is a freely available web server that allows investigation of the association between estimated immune infiltration and gene expression [100]. We employed TIMER 2.0 to determine the correlation between the expression of key lectins and immune infiltration in TCGA-GB macrophages, myeloid dendritic cells, CD4^+^ T cells, CD8^+^ T cells and neutrophils. MDSCs and Tregs were not included due to unavailability of TIMER algorithm analysis. The purity-adjusted Spearman’s rho was determined for the different cell types, as purity has a negative correlation with immune cell type.

### 5.6. Patients’ Tumor Biopsies and Blood Sample Processing

Patient-matched tumor biopsies and peripheral blood samples were obtained from 20 patients from the Neurology and Neurosurgery department at Policlinico Umberto I Hospital (Rome, Italy) (Supplementary Table S3). All samples were collected in accordance with the Declaration of Helsinki and with good clinical practice guidelines, and all patients signed informed consent forms (Policlinico Umberto I Ethics Committee Protocol, RIF.CE: 4181). Resected tumor samples were processed freshly within 2 h from collection. Tissue samples were first mechanically dissected under sterile conditions. For enzymatic dissection, the tumor suspension was incubated at 37 °C with 5% CO_2_ for 45 min in RPMI supplemented with 10% fetal bovine serum (FBS), DNAse (10 μg/mL) and Collagenase (1 mg/mL) solution. The tumor suspension was then filtered through a 100 µm cell strainer and centrifuged at 1500 rpm for 8 min at 4 °C to pellet the cells and myelin mixture. Then, myelin removal was performed by gradient centrifugation with 30% Percoll (Sigma-Aldrich, St. Louis, MO, USA) in 1× Phosphate-Buffered Saline (PBS) at 3000 rpm for 30 min at 4 °C. Finally, the single-cell suspension was washed two times in 1× PBS at 1500 rpm for 8 min at 4 °C to pellet cells. Cells were then used for downstream analysis. Peripheral blood samples were stratified on a Ficoll-Hypaque gradient (1077 g/mL; Lympholyte-H, Cedarlane, Burlington, VT, Canada). Density centrifugation was performed at room temperature at 1800 rpm for 30 min with no brake. Peripheral blood mononuclear cells (PBMCs) were collected, washed three times in PBS 1× and analyzed as described below.

### 5.7. Lectin Expression on Tumor-Infiltrating and Circulating Myeloid Cell Subsets

Cells obtained from matched tumor samples and blood samples were used for the characterization of ASGR2 and CLEC12A lectin expression on immune cell populations (total CD45, macrophages, microglia, monocytes, M-MDSCs, LOX1^+^ PMN-MDSCs, neutrophils) by flow-cytometry analysis (FC). The following fluorochrome-conjugated antibodies were used: CD45-KrO, CD15-PB, CD14-ECD and CD66b-APC-A750 (Beckman Coulter, Brea, CA, USA); P2RY12-BV421, CD49d-PE, CLEC12A-PECy7, LOX1-PE, and CD11c-PerCP/Cy5.5 (BioLegend, San Diego, CA, USA); ASGR2-AF700 (R&D System, Minneapolis, MN, USA). Yellow fluorescent reactive dye (Invitrogen, Waltham, MA, USA) was used to discriminate between viable and dead cells. To ensure accurate positive signals and the gating strategy for both lectins, Fc Block (BD Biosciences, San Jose, CA, USA) was used in each sample to prevent non-specific antibodies from binding, and Fluorescence minus one (FMO) and autofluorescence were further used as negative controls. Briefly, 1 × 10^6^ cells were distributed in polypropylene tubes and incubated with the antibody mixture for 30 min at room temperature and then washed in 1× PBS at 1200 rpm for 5 min. Cells were then acquired by DxFlex Flow Cytometer (Beckman-Coulter, Brea, CA, USA), and analysis was performed with FlowJo 10th software (Becton Dickinson, San Jose, CA, USA). Data for CD45^+^ cells are reported as percentage of live cells. Data for myeloid cell subsets are reported as percentage of total CD45^+^ infiltrating and circulating immune cells.

### 5.8. Statistical Analysis

Statistical analysis of flow-cytometric data was performed with GraphPad Prism version 8 (Graphpad Software, Inc., San Diego, USA). Descriptive statistics [average and standard error media (SEM)] were used to describe the various data. ANOVA (Analysis of Variance) followed by Tukey’s multiple comparison test was used to compare means across multiple groups. The Kaplan–Meier method was used to calculate overall survival (OS). Differences between Kaplan–Meier curves of OS were assessed using the log-rank test. The results with *p* values < 0.05 were considered statistically significant: * *p* < 0.05, ** *p* < 0.01, *** *p* < 0.001, **** *p* < 0.0001.

## Data Availability

Data is provided within the manuscript or Supplementary Information files.

## Supplementary Materials

The following supporting information can be downloaded at: https://www.mdpi.com/article/10.3390/ijms27062626/s1.

## Abbreviations

The following abbreviations are used in this manuscript:

GB	glioblastoma
WHO	World Health Organization
IDH	isocitrate dehydrogenase
BBB	blood–brain barrier
MDSCs	myeloid-derived suppressor cells
TIME	tumor immune microenvironment
TAMs	tumor-associated macrophages
ECM	extracellular matrix
Tregs	regulatory T cells
ARG1	arginase 1
ROS	reactive oxygen species
CRD	specialized recognition domain
Siglecs	sialic binding lectin receptors
TCGA	the Cancer Genome Atlas Program
GTEx	genotype-Tissue Expression
CGGA	chinese Glioma Genome Atlas
COPD	chronic obstructive pulmonary disease
PD	Parkinson’s disease
AML	acute myeloid leukemia
MG	resident microglia
CNS	central nervous system
EAE	experimental autoimmune encephalomyelitis
DCs	dendritic cells
TIMER 2.0	tumor Immune Estimation Resource 2.0
FBS	fetal bovine serum
PBS	phosphate-buffered saline
FC	flow-cytometry analysis
FMO	fluorescence minus one
SEM	standard error media
ANOVA	analysis of variance
OS	overall-survival
