# Supplementary material for: ASGR2 and CLEC12A as Prognostically Relevant C-Type Lectin Hubs in Glioblastoma
Source: Int J Mol Sci. 2026 Mar 13;27(6):2626. doi: 10.3390/ijms27062626 (PMC13026556; doi:10.3390/ijms27062626)
Supplement: Supplementary file 1 [file ijms-27-02626-s001.zip › ijms-4150800-supplementary.pdf]

Supplementary Table S1 – Fold change of lectins in TCGAvsGTEx differential co-expression network

Gene	FoldChange	P value
CLEC10A	2.22	1.25e-06
LGALS2	2.75	5.53e-06
LGALS3	1.51	5.40e-12
LGALS3BP	2.58	3.14e-38
LGALS4	-1.00	3.14e-07
LGALS8	-0.54	5.48e-16
LGALS9	2.44	5.48e-32
CD22	-4.19	5.42e-28
CD33	3.49	2.49e-30
MAG	-1.97	3.44e-09
SIGLEC5	0.59	3.74e-09
SIGLEC7	4.91	4.72e-24
SIGLEC8	3.15	2.83e-23
SIGLEC9	3.88	1.82e-22
SIGLEC10	2.88	1.23e-22
SIGLEC11	1.20	1.46e-19
ACAN	0.66	9.14e-04
BCAN	1.86	2.66e-09
NCAN	2.81	2.01e-16
VCAN	2.21	9.30e-30
FREM1	-0.36	1.10e-01
CD209	0.85	1.25e-04
CLEC4A	1.13	5.08e-16
ASGR2	-1.28	3.38e-18
CD207	2.57	4.28e-07
CLEC4E	2.16	1.19e-10
CLEC5A	5.65	1.85e-25
CLEC2B	1.59	1.39e-10
CLEC1A	0.13	9.04e-01
CLEC1L	3.50	4.94e-21

CLEC12A	3.07	1.10e-15
CLEC9A	0.59	8.97e-01
CLEC7A	2.07	3.07e-20
CLEC2D	0.55	2.92e-11
CLEC11A	0.96	2.94e-01
CLEC3B	-0.68	9.37e-01
CLEC14A	0.11	8.12e-01
CLEC18A	0.83	1.60e-08
CLEC17A	2.81	8.08e-17

Supplementary Table S2: List of total and filtered lectins

Lectins	Gene symbol	TCGA		CGGA		GTEX	
		Available	Filtered	Available	Filtered	Available	filtered
Galectin 1	LGALS1	-		+			
Galectin 2	LGALS2	+	+	+	+		+
Galectin 3	LGALS3	+	+	+	+		+
Galectin 3 binding protein	LGALS3BP	+	+	+	+		+
Galectin 4	LGALS4	+	+	+	+		+
Galectin 7	LGALS7	+		+			
Galectin 8	LGALS8	+	+	+	+		+
Galectin 9	LGALS9	+	+	+	+		+
Galectin 9B	LGALS9B	+		+			
Galectin 9C	LGALS9C	+		+			
Galectin 10	LGALS10	-		+			
Galectin 12	LGALS12	-		+			
Galectin 13	LGALS13	+		+			
Galectin 14	LGALS14	-		+			
Galectin 16	LGALS16	+		+			
Siglec-1	SIGLEC1	-		+			
Siglec-2	CD22	+	+	+	+		+
Siglec-3	CD33	+	+	+	+		+
Siglec-4a,	MAG	+	+	+	+		+
Siglec-5	SIGLEC5	+	+	-			+
Siglec-6	SIGLEC6	+		+			
Siglec-7	SIGLEC7	+	+	+	+		+
Siglec-8	SIGLEC8	+	+	+			+
Siglec-9	SIGLEC9	+	+	+	+		+

Siglec-10	SIGLEC10	+	+	+	+	+
Siglec-11	SIGLEC11	+	+	+	+	+
Siglec-14	SIGLEC14	-		+	+	
Siglec-15	SIGLEC15	+		+		
Siglec-16	SIGLEC16	-		+	+	
Aggrecan	ACAN	+	+	+	+	+
Brevican	BCAN	+	+	+		+
Neurocan	NCAN	+	+	+		+
Versican	VCAN	+	+	+		+
FRAS1 related extracellular matrix 1	FREM1	+	+	+		+
Blood Dendritic Cell Antigen 2	CLEC4C	+		+		
DC-SIGN	CD209	+	+	+		+
DC-SIGN2	CLEC4M	+		+		
Dectin-2	CLEC6A	+		+		
Dendritic cell immunoreceptor (DCIR)	CLEC4A	+	+	+	+	+
Fc fragment of IgE receptor II	FCER2	+		+	+	
Hepatic Asialoglycoprotein Receptor 1	ASGR1	-		+	+	
Hepatic Asialoglycoprotein Receptor 2	ASGR2	+	+	+	+	+
Kupffer Cell receptor	CLEC4F	-		+		
Langerin	CD207	+	+	+		+
Liver sinusoidal epithelial cell lectin (LSECtin)	CLEC4G	+		+		
Macrophage galactose-type lectin (MGL)	CLEC10A	+	+	+	+	+
Macrophage C-type Lectin (MCL)	CLEC4D	+		+	+	
MINCLE	CLEC4E	+	+	+	+	+
C-type lectin domain family 2 member L	CLEC2L	-		+	+	
C-type lectin domain containing 5A	CLEC5A	+	+	+		+
C-type lectin domain family 2 member B	CLEC2B	+	+	+	+	+
C-type lectin domain family 1 member A	CLEC1A	+	+	+	+	+
C-type lectin domain family 1 member B	CLEC1B	+		+		
C-type lectin domain family 12 member B	CLEC12B	+		+		
C-type lectin-like 1	CLECL1	+	+	+		+

C-type lectin domain family 12 member A	CLEC12A	+	+	+		+
DNGR	CLEC9A	+	+	+		+
C-type lectin domain family 2 member A	CLEC2A	+		+		
Dectin-1	CLEC7A	+	+	+		+
C-type lectin domain family 2 member D	CLEC2D	+	+	+		+
C-type lectin domain containing 19A	CLEC19A	+		+	+	
Cartilage-derived C-type lectin	CLEC3A	+		+	+	
Stem cell growth factor (SCGF)	CLEC11A	+	+	+	+	+
Tetranectin	CLEC3B	+	+	+	+	+
C-type lectin domain containing 14A	CLEC14A	+	+	+		+
C-type lectin domain family 18 member A	CLEC18A	+	+	+	+	+
Prolectin	CLEC17A	+	+	+		+

Supplementary Table S3: total number of cases used in the study.

Database	Available	Selected	Total
**TCGA**	152	145	145
**CGGA**	133	133	133
**GTEx**	255	255	255
**Biological samples**			
**Blood**			20
**Tumor**			20

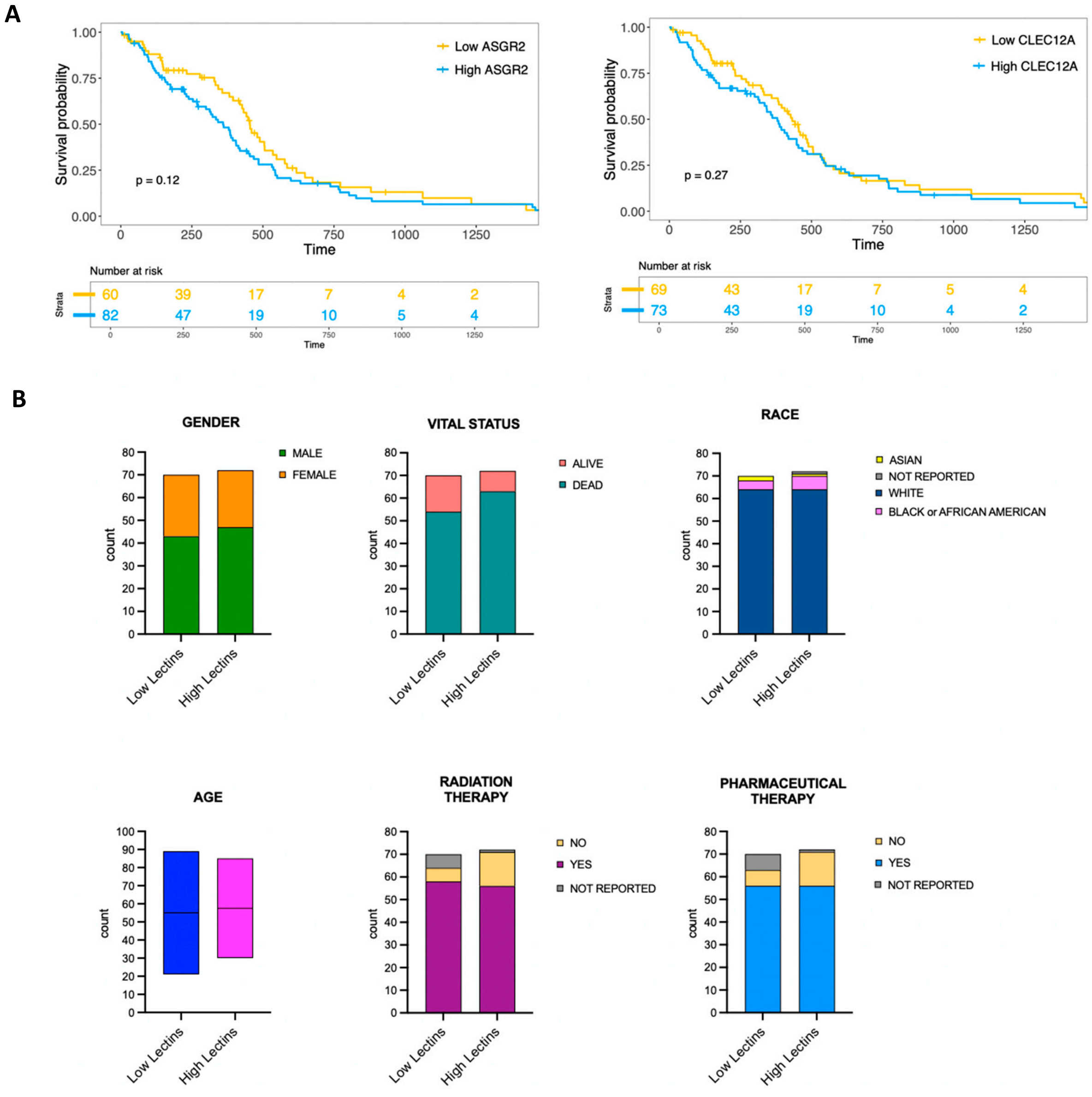

Figure S1- Survival curves and Clinical features of the patients' clusters. A) Kaplan-Meier survival curves of GB patients distributed in the two cohorts based on ASGR2 and CLEC12A expression levels. Log-rank test was employed to determine the statistical significance of observed difference [ASGR2^{high} *vs* ASGR2^{low} (p=0.12) and CLEC12A^{high} *vs* CLEC12A^{low} (p=0.27)]. B) Histograms represent the differences in clinical features and parameters (age, vital status, race, gender, type of therapy) between the two cluster of patients (high lectins vs low lectins) identified by Fuzzy C-mean clustering method for ASGR2 and CLEC12A co-expression.

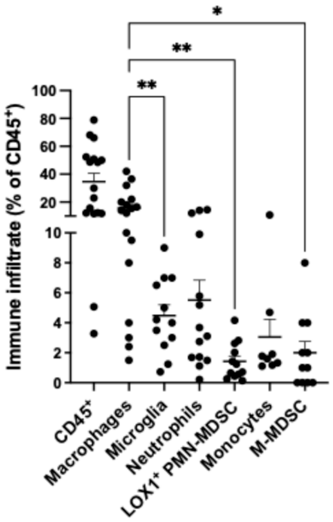

Figure S2 – Immune infiltrate contribution in GB samples. A) Scatter dot-plot represent the immune infiltrate composition in GB samples (total CD45, macrophages, microglia, LOX1^{+} PMN- and M-MDSC, neutrophils, monocytes). Each myeloid cell population was analyzed by multiparametric analysis by flow-cytometry. Data of myeloid cell subsets are reported as mean ± SEM and as percentage of CD45^{+} total immune infiltrating cells. Data of CD45^{+} cells are reported as percentage of live cells. ANOVA followed by Tukey's multiple comparison test was used to evaluate differences among different myeloid subset. *, $p<0.05$ was considered statistically significant.

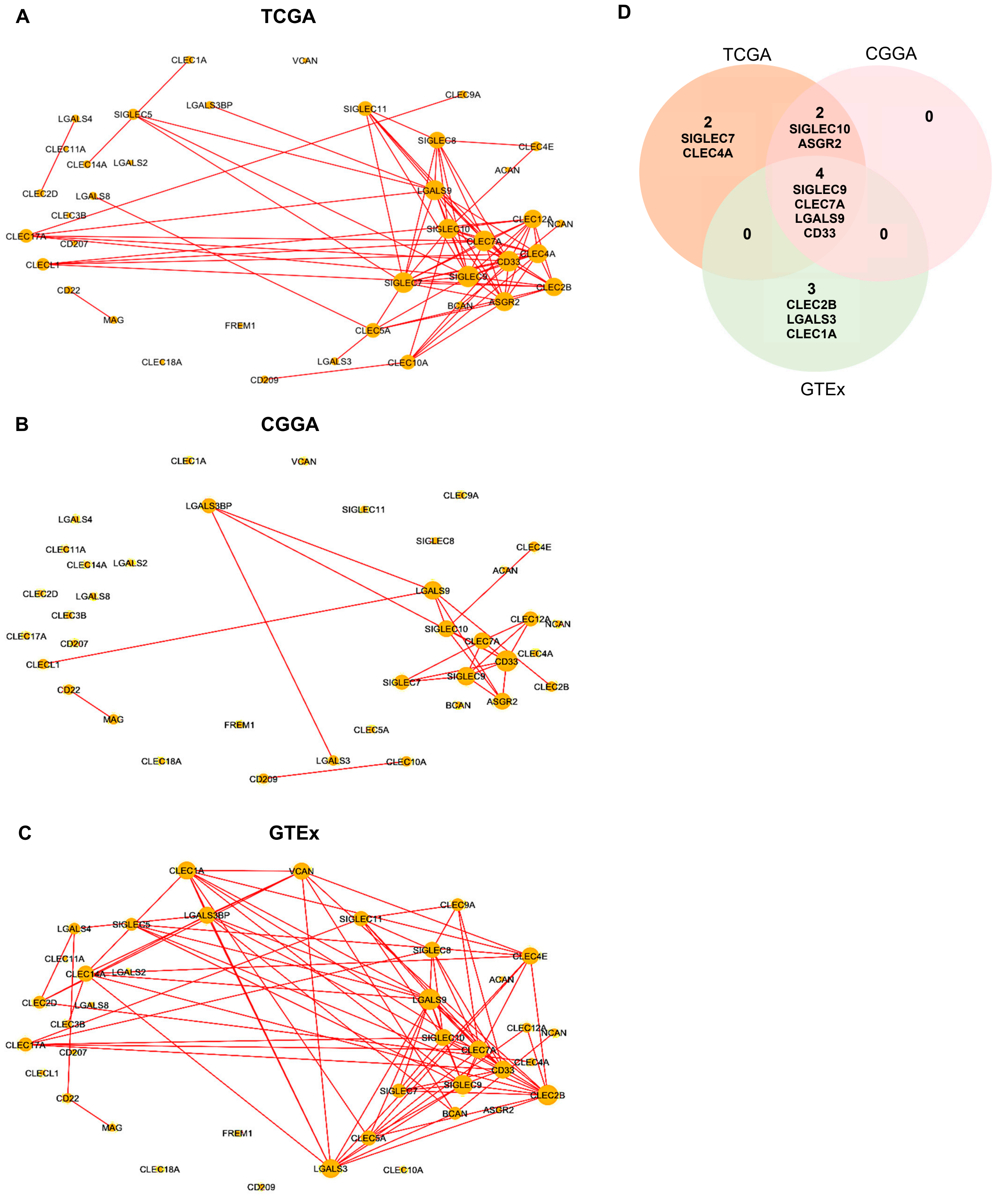

Figure S3- Co-expression networks of glioblastoma and healthy brain tissue. A) Co-expression network of TCGA. B) Co-expression network of CGGA.C) Co-expression network of GTEx. D) Venn diagram showing the degree of agreement between the three co-expression networks with respect to the sets of hub lectins.
